# Exploring Endothelial Expansion on a Chip

**DOI:** 10.3390/s22239414

**Published:** 2022-12-02

**Authors:** Joanna Konopka, Dominik Kołodziejek, Magdalena Flont, Agnieszka Żuchowska, Elżbieta Jastrzębska, Zbigniew Brzózka

**Affiliations:** 1 Faculty of Chemistry, Warsaw University of Technology, 00-661 Warszawa, Poland; 2 Centre for Advanced Materials and Technologies CEZAMAT, Warsaw University of Technology, 02-822 Warszawa, Poland

**Keywords:** angiogenesis-on-a-chip, vessel-on-a-chip, sprouting, Viscous Finger Patterning (VFP), micro-milling, Vascular Endothelial Growth Factor (VEGF), Human Umbilical Vein Endothelial Cells (HUVECs), lumen, microvessel, microfluidics

## Abstract

Angiogenesis is the development of new blood vessels from the existing vasculature. Its malfunction leads to the development of cancers and cardiovascular diseases qualified by the WHO as a leading cause of death worldwide. A better understanding of mechanisms regulating physiological and pathological angiogenesis will potentially contribute to developing more effective treatments for those urgent issues. Therefore, the main goal of the following study was to design and manufacture an angiogenesis-on-a-chip microplatform, including cylindrical microvessels created by Viscous Finger Patterning (VFP) technique and seeded with HUVECs. While optimizing the VFP procedure, we have observed that lumen’s diameter decreases with a diminution of the droplet’s volume. The influence of Vascular Endothelial Growth Factor (VEGF) with a concentration of 5, 25, 50, and 100 ng/mL on the migration of HUVECs was assessed. VEGF’s solution with concentrations varying from 5 to 50 ng/mL reveals high angiogenic potential. The spatial arrangement of cells and their morphology were visualized by fluorescence and confocal microscopy. Migration of HUVECs toward loaded angiogenic stimuli has been initiated after overnight incubation. This research is the basis for developing more complex vascularized multi-organ-on-a-chip microsystems that could potentially be used for drug screening.

## 1. Introduction

The physiological process of creating new blood vessels is based on three main mechanisms: vascularization, angiogenesis, and arteriogenesis. Vascularization is defined as the de novo synthesis of blood vessels. It mainly occurs during embryonic development, and it results in primary vascular plexus formation. The primary plexus grows into more complex networks throughout a process called angiogenesis (aka neovascularization) [1]. Under physiological conditions, angiogenesis is involved in multiple processes such as wound healing [2], placenta formation, growth of the endometrium [3], cyclical growth of hair and follicles [4], and the weight gain of trained skeletal muscles [5]. More precisely, the process occurs in poorly supplied oxygen and other nutrient tissue sites. One of the most common angiogenic mechanisms is called sprouting. Sprouting angiogenesis relies on the branching of blood vessels beyond the lumen (i.e., perfusable space surrounded by ECs within which blood flows). As malnutrition of parenchymal cells occurs, Vascular Endothelial Growth Factor (VEGF) is released and an angiogenic cascade is launched. Consequently, endothelial cells (ECs) migrate and proliferate within the extra-cellular matrix (ECM) toward an angiogenic stimulus. When two tip cells merge, a new vascular structure is formed, and it is stabilized due to the pericyte’s action [6].

Many pro- and anti-angiogenic factors control the course of the angiogenesis process. Disorders of the distribution of these agents lead to the development of multiple diseases. Specifically, overexpression of pro-angiogenic factors contributes to the uncontrolled proliferation of endothelial cells that is observed in neoplastic diseases, rheumatoid arthritis, and in multiple sclerosis, while increased synthesis of anti-angiogenic factors results in the development of osteoporosis, ulcers, and cardiovascular diseases, for instance [7,8]. Both cardiovascular diseases and cancers are qualified by the World Health Organization (WHO) as the leading cause of death worldwide [9,10]. A better understanding of the mechanisms regulating physiological and pathological angiogenesis will potentially contribute to developing more effective treatments for those urgent issues. Therefore, various attempts have been made to recapitulate and analyze the angiogenesis process. Two-dimensional cell cultures, commonly used in pre-clinical research, fail to faithfully mimic neovascularization [11,12,13,14]. Hence, microfluidic devices, known as angiogenesis-on-a-chip, have emerged. Microfluidic conditions allow for the analysis of (i) intercellular interactions; (ii) interactions of cells with extracellular matrix; and (iii) drug distribution through endothelial barrier [15]. Faithfull simulation of angiogenesis under in vitro conditions requires the simultaneous application of various biochemical and biophysical factors [16,17,18,19]. A key agent that determines the initiation of angiogenesis is Vascular Endothelial Growth Factor (VEGF) [20,21]. It has already been successfully used to initiate angiogenesis in microfluidics [22,23,24,25]. Its concentration can be regulated directly or indirectly. In the case of direct regulation, protein is dosed directly into the flowing medium. It has been observed that its low concentrations stimulate the migration of ECs the most efficiently. However, what is hidden under the term “low” remains unclear. Some suggest the use of VEGF solution with a concentration starting from 2.5–5 ng/mL [26], while others with a concentration of 50 ng/mL [22] or even 100–500 ng/mL [27]. 

Furthermore, VEGF can be delivered indirectly by (i) a diminution in oxygen pressure, (ii) an increase in lactate concentration, (iii) an increase in bioactive amines concentration, and by (iv) some types of cells such as macrophages, fibroblasts, keratinocytes, platelets and tumor cells [27,28,29,30]. Angiogenesis plays a key role in the process of tumorigenesis and cancer metastasis. Thus, one of the most often examined co-cultures consists of tumor spheroids and endothelial cells. Furthermore, the pro-angiogenic activity of tumor spheroids can be enhanced by the addition of stromal cells such as fibroblasts [31,32]. 

Numerous promising studies on angiogenesis have been effected in microfluidics. However, a fully functional microplatform has not yet been engineered. As new tools for angiogenesis studies are required, our research focused on (i) reconstruction of the luminal structure of blood vessels in vitro, (ii) design and manufacture of a microfluidic cell culture system called angiogenesis-on-a-chip, and (iii) initiation of angiogenesis under microfluidic conditions. More precisely, we have developed a microfluidic chip that allowed us to reconstruct the three-dimensional structure of primary blood vessels using the Viscous Finger Patterning technique. We have optimized the VFP procedure toward the repetitive creation of lumens with different diameters. As there is no clear answer to which concentration of VEGF should be used to successfully initiate angiogenesis, the main objective of the study was to investigate the influence of different concentrations of VEGF on HUVECs migration under coherent conditions. To the best of our knowledge, those two aspects have not been previously studied.

## 2. Materials and Methods

### 2.1. Cell Culture

Human Umbilical Vein Endothelial Cells (HUVECs) were sourced from (Lonza, Basel, Switzerland,). They were cultured in Endothelial Cell Growth Medium-2 (EGM-2, PromoCell, C-22211, Heidelberg, Germany) at 37 °C and 5% CO_2_ up to 8 passages. HUVECs were centrifuged at 1000 RPMI for 5 min during passaging to remove trypsin solution. 

### 2.2. Manufacture of a Microplatform

**Figure 1:**
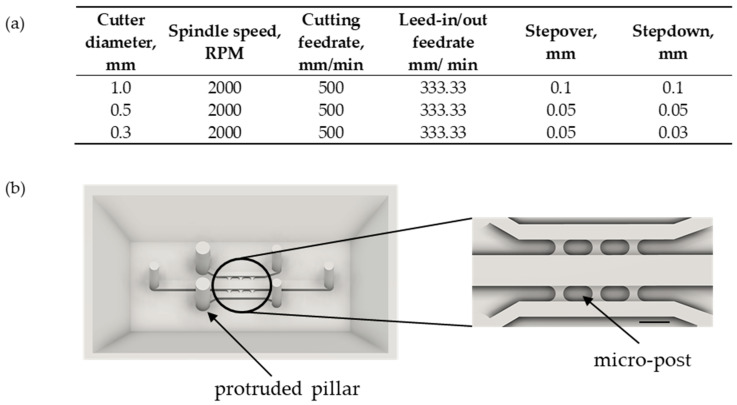
(**a**) Micro-milling parameters. (**b**) Exemplary mold with protruded pillars. Scale bar: 1000 µm.

Microplatform consists of two layers: an upper layer made of poli(dimethylsiloxane) (PDMS) within which microstructures are patterned and a bottom layer—a microscopic cover glass (170 ± 5 µm thick) that acts as a sealing. PDMS layer is made with the use of a soft lithography technique either using micro-milling technique in a poli(methyl(methacrylate)) (PMMA) plaque or by 3D printing using grey standard resin (Flashforge, FH1100, Warsaw, Poland) following producer’s instructions. The operating parameters of the micro-milling process are summarized below (Figure 1a). Three end mills with diameters of 1.0 mm, 0.5 mm, and 0.3 mm were used for the process. The cutter with the largest diameter was used to rough PMMA plaque, then the smaller cutters were used to smooth its surface and remove the remaining material between the microchannels. Qualitative and quantitative analysis of the accuracy of the fabrication process was carried out with the use of 3D Laser Measuring Microscope (LEXT Olympus4000, Tokyo, Japan, Table 1). More precisely, the images of crucial microstructures were captured and analyzed. Then, the dimensions of the digital design with its execution were compared. The designed mold includes protruded pillars (Figure 1b) to avoid punching holes. The ready-to-use mold was filled with non-cross-linked PDMS prepolymer solution mixed with a cross-linking agent in a weight ratio of 10:1 (Sylgard Silicone elastomer 184, Steinfurt, Germany). The prepolymers mixture was thoroughly degassed in a desiccator. Then, a mold was incubated at 65 °C for 1 h. Next, the PDMS cast was removed from a mold, degreased, rinsed with distilled water, and allowed to dry completely. Finally, the PDMS layer was bonded with a coverslip glass with an oxygen plasma generator (time: 30 s, power: 80%, Diener ATTO). Microplatform was left under load for 24 h to ensure efficient bonding. The geometry and dimensions of tested microplatforms are described in detail in the following sections. 

### 2.3. Lumen’s Formation

Lumens were created with the use of Viscous Finger Patterning (VFP) technique [31]. First, the surface of the microchannels was modified to ensure good adhesion of the collagen matrix. More precisely, microchannels were filled with a 2 mg/mL solution of polydopamine (Sigma-Aldrich, H8502, Burlington, MA, USA) in Tris buffer with final pH of 8.5 (Sigma Aldrich, T1503). Microplatforms were then incubated at room temperature (RT) for 1 h. After the incubation, polydopamine solution was washed out by miliQ water. Then, microchips were dried for min. 1 h at 65 °C. Next, collagen type I solution with the final concentration of 5 mg/mL and pH = 7–8 was prepared by mixing PBS (1X) (Sigma-Aldrich, P54931L, USA), PBS (10×) (Gibco, 70011044, New York, NY, USA), 1 M NaOH (Sigma-Aldrich, S2770, Burlington, MA, USA) and rat tail collagen type I with a concentration of 9.48 mg/mL (Corning, 354249, New York, NY, USA). Volumes of reagents were determined according to producer’s instructions.

Cold and well-mixed solution of collagen type I was introduced into rectangular microchannels using an automatic pipette until the microchannel was filled. Then, a single droplet of phosphate-buffered saline solution (PBS(1X)) was placed on the surface of the outlet, which is a hole with a greater diameter (1.5 mm). Next, small droplets were introduced into the inlet of the microchannel. Consequently, a pressure difference was created, thanks to which PBS flow occurred. Therefore, a cylindrical microchannel surrounded by a thin layer of collagen matrix was formed. Microplatforms were incubated at 37° for min 30 min to ensure hydrogel cross-linking. After that, a cylindrical lumen was ready for endothelial cell seeding. The whole procedure was performed under sterile conditions and using sterile reagents. 

### 2.4. HUVECs Loading and Its Culture within a Microfluidic Platform

HUVECs were loaded twice (10^7^ cells per microchannel). The second loading was followed by a turnover of a microplatform by 180° (see optimization studies in Section 3.1.2.). Every day, microchannels were rinsed with EGM-2. The microchips were placed in a Petri dish containing a smaller one filled with PBS(1X) solution to minimalize the risk of drying the inlets.

### 2.5. Pro-Angiogenic Agent Loading

Pro-angiogenic agent was loaded into the central microchannel with the use of an automatic pipette. Firstly, VEGF_165_ (Sigma-Aldrich, USA) with different concentration values (5, 25, 50, 100 ng/mL) was used. Every day, solution within a central microchannel was replaced by a fresh one. Cell culture within a microplatform was maintained for no longer than 10 days.

### 2.6. Analysis of Cell Behavior and Their Morphology

Analysis of the spatial arrangement of endothelial cells and their morphology was performed every day with the use of fluorescence microscopy (Olympus, IX71, Tokyo, Japan). Captured images were analyzed with the use of computer software to ensure quantitative results as well. Furthermore, immunostaining of loaded cells was performed on the last day of cell culture and it was followed by confocal microscopy observations (Olympus FluoView FV10i or Zeiss Axio Observer 7). Immunostaining was carried out as follows. Firstly, all the microchannels were rinsed with PBS(1X) and then 4% formaldehyde (Sigma-Aldrich, 158127, USA) was introduced into them. Microplatforms were then incubated for 30 min at RT. After the incubation, microchannels were washed with PBS(1X), filled with PBS(1X) containing 0.1% Triton X-100 (Sigma-Aldrich, T8787, USA), and incubated for 20 min at RT. After that time, microchannels were rinsed with the use of PBS(1X), then filled with PBS(1X) containing 1% BSA, and incubated for 1 h at RT. Afterward, microchannels were filled with a solution of 1% BSA (Sigma-Aldrich, A7906, USA) containing Hoechst (1:100) (Invitrogen) and phalloidin (1:400) (Invitrogen). Microplatforms were incubated for 1 h at RT, washed out with PBS(1X) leaving PBS(1X) solution within microchannels. 

### 2.7. Statistical Analysis

At least three independent experiments were performed for each measurement (n ≥ 3). The obtained results were averaged, and the standard deviation (SD) was determined. In addition, a one-way analysis of variance was performed using the ANOVA (analysis of variance) test.

## 3. Results

### 3.1. Vascularization under Microflow Conditions

#### 3.1.1. Lumen Formation

**Figure 2:**
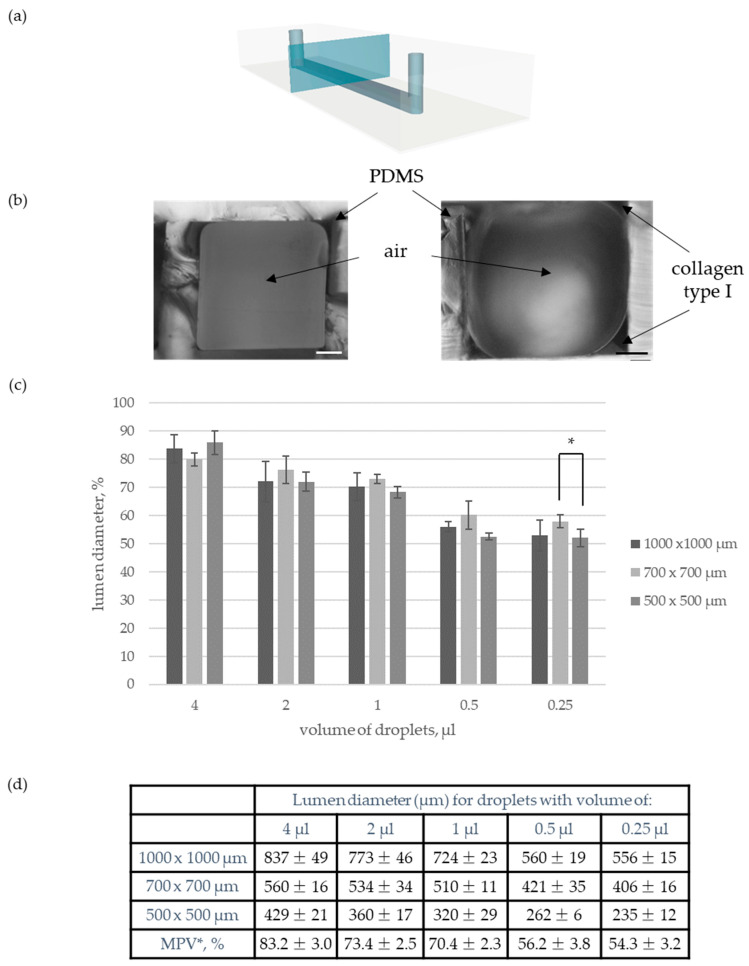
(**a**) Schematic view of single microchannel with marked section plane used for following photos. (**b**) Microchannel’s cross-section before and after modification by VFP. Scale bar: 200 µm. (**b**) Influence of droplet’s volume on lumen diameter. (**c**) Mean values of lumen diameter. * Mean percentage value of rectangular microchannel’s width covered by lumen diameter.

Under physiological conditions, blood vessels have luminal structures. It is essential to consider that aspect while wishing to create a faithful tissue model. Thus, the modified Viscous Finger Patterning (VFP) technique developed by Bischel et al. was applied to described studies [31]. VFP is based on the hydrodynamic phenomenon that relies on a displacement of a viscous fluid by another one with lower viscosity. Consequently, a rectangular microchannel was transformed into a cylindrical one. The edges of a modified microstructure were filled with collagen matrix while its interior, called lumen, remained hollow and perfusable. A comparison of microchannel cross-sections (Figure 1a) before and after the described modification is shown in (Figure 2b).

Afterward, the diameter of the lumen was adjusted to desired parameters. For that purpose, the influence of the volume of dispensed droplets on the diameter of the cylindrical microchannel was studied. More precisely, five different dosing models were tested within three straight rectangular microchannels differing in dimensions: (i) 1000 µm × 1000 µm, (ii) 700 µm × 700 µm, and (iii) 500 µm × 500 µm. Specifically, a droplet of 20 µL was introduced on the outlet (Ø 1.5 mm), and small droplets of either 4 µL, 2 µL, 1 µL, 0.5 µL or 0.25 µL were introduced into the inlet (Ø 1.0 mm) with the use of an automatic pipette. It has been observed that with the decrease in the dispensed droplets volume, the microvessel’s diameter decreased (Figure 2c and Figure S1). This phenomenon is because smaller droplets spread faster along the hydrogel matrix than larger ones. Moreover, it has been observed that the lumen diameter covers an analogous percentage of the rectangular microchannel width for every dosing model. The validity of the hypothesis was confirmed by one-way ANOVA analysis. Therefore, mean percentage values (MPV) for every dosing pattern were determined. Established values allow easy adjustment of the VFP procedure for desired lumen diameter (Figure 2d). 

#### 3.1.2. HUVECs Loading

**Figure 3:**
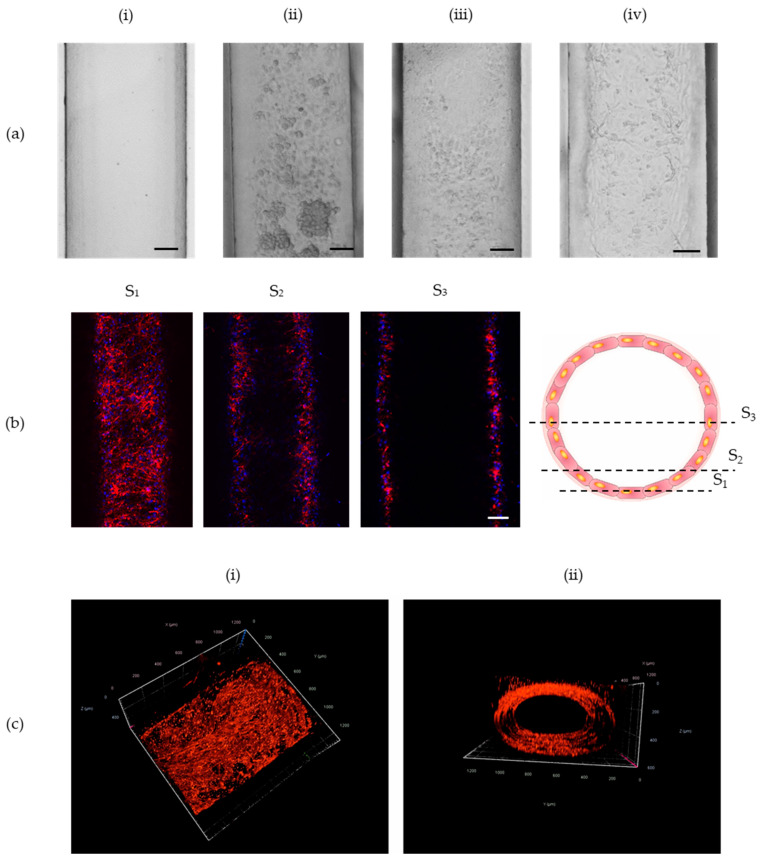
Structure of microvessel created by Viscous Finger Patterning. (**a**) Images present respectively cross-section of a microchannel (**i**) before cell’s loading (**ii**) seeded with $2\cdot 10^{7}\mathrm{of}\;\mathrm{HUVEC}\;\mathrm{cells}$ and constantly rotated for 2 h by 360°, (**iii**) seeded with $2\cdot 10^{7}\mathrm{of}\;\mathrm{HUVEC}\;\mathrm{cells}$ and rotated every 15 min by 90° (**iv**) seeded twice with $10^{7}\mathrm{of}\;\mathrm{HUVEC}\;\mathrm{cells}$ and rotated only once by 180° after the second loading of cells. Scale bar: 100 µm (**b**) Chosen frames from z-stack image. Cells were stained with the use of phalloidin and Hoechst. Scale bar: 100 µm (**c**) 3D structure of created microvessel: (**i**) top view, (**ii**) front view.

Subsequently, optimizing studies aiming at homogenous adhesion of endothelial cells over a cylindrical microstructure were performed. Three different approaches of incubating endothelial cells directly after loading into the microchannel were examined. Firstly, cells were introduced once, and then the microplatform was rotated by 360° for two h with the use of a spinning wheel (Figure S2) that rotated at a speed of six turns per minute. This solution caused aggregation of cells, and it did not allow for their homogenous adhesion into the walls (Figure 3(aii)). Therefore, it has been decided to rotate a microplatform every 15 min by 90° to ensure more time for cell adhesion. That solution was successful. However, it was very time-consuming. Therefore, an alternative method that relied on a double introduction of cell suspension was used. The microplatform was rotated only once by 180° directly after the second loading of cells (i.e., two h after the first loading of HUVECs). This approach allowed for the homogenous adhesion of cells around the cylindrical microstructure. Moreover, it is the easiest and fastest solution among those tested. A comparison of an empty channel and HUVECs adhesion into its walls is presented in Figure 3a. To observe the 3D structure of the created microvessel, z-stack images were taken with the use of confocal microscopy. Prior to taking z-stack images, the cells were stained with the use of phalloidin and Hoechst. Chosen frames from the 3D structure of a microvessel are shown in Figure 3b. Figure 3c presents the integrity of a microvessel’s cylindrical structure.

### 3.2. Angiogenesis under Microflow Conditions

#### 3.2.1. Design and Manufacture of a Microplatform

**Figure 4:**
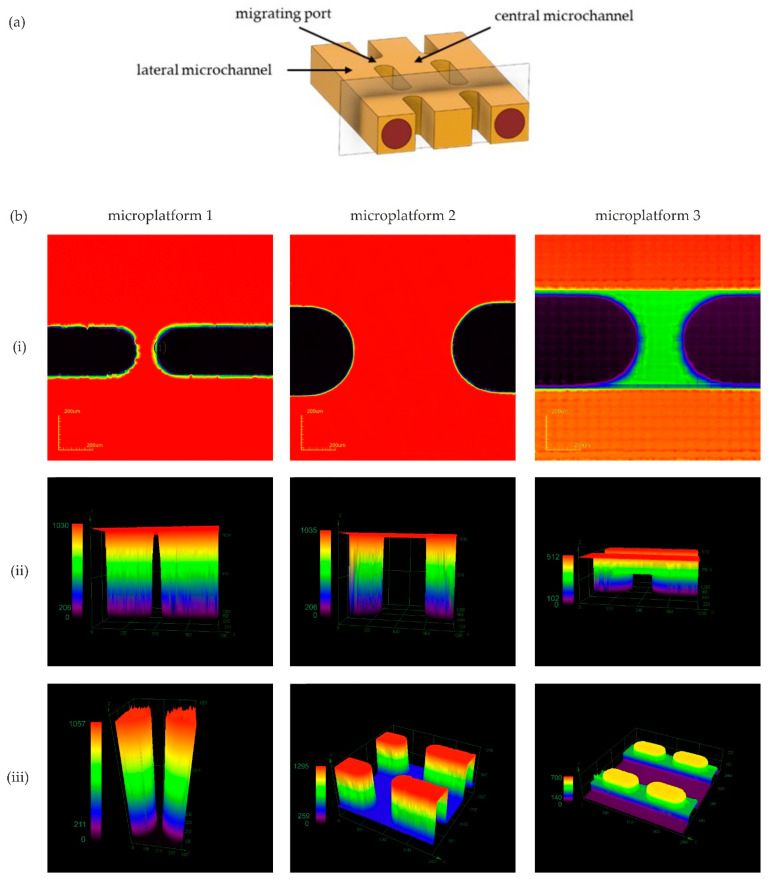
(**a**) Cross-sectional view of desired configuration of microchannels with marked section plane used for photos of (**iii**) PDMS cast. (**b**) Following images show: (**i**) top view of micro-milled migrating ports (PMMA mold), (**ii**) comparison of height of migrating ports (PMMA mold), (**iii**) images of migrating ports cast in PDMS. All images were captured with the use of 3D Laser Measuring Microscope (LEXT Olympus4000).

The basic concept of a microplatform assumes a projection of three parallel microchannels. Two lateral ones are designed for the formation of cylindrical microvessels, while the central microchannel allows the loading of an angiogenic factor (Figure 4a). Those three main microchannels relate to each other by migrating ports which provide observation of angiogenic migration of endothelial cells within sprouting. While adjusting the dimensions of a microplatform, it was essential to consider the controlled spread of the collagen matrix. More precisely, hydrogel should fill only the lateral microchannels and migrating ports, steering clear of the central microchannel. All the designs described below differ only in the dimensions of migrating ports.

First, migrating ports with the smallest possible width (50 µm) provided by our equipment were designed. This project was executed with the use of the micro-milling technique. The analysis of images captured by a 3D Laser Measuring Microscope proved that such narrow microstructures have been successfully patterned (Figure 4b). Moreover, the collagen solution did not enter the central microchannel as desired. However, such a small area of the migrating ports did not allow observations of angiogenic sprouting. Therefore, migrating ports were expanded. The second version of the microchip geometry included migrating ports with a significantly greater width of 500 µm (Figure 4c). Such an invasive change has resulted in a loss of control over the spread of the collagen matrix, and indeed hydrogel solution has filled the whole microsystem. Our findings are consistent with previous studies according to which the distance between micro-posts cannot exceed 200 µm to control the liquid’s spread [14]. Finally, controlled hydrogel distribution was obtained due to a diminution in the height of migrating ports (Figure 4d). More precisely, the height of migrating ports is equal to half of the height of the lateral microchannels (500 µm) and a quarter of the central microchannel (1000 µm), which is 250 µm. Such a model has an added benefit. It allows the creation of a vascular lumen not only in lateral channels but in a central microchannel as well. Consequently, two different angiogenic agents may be loaded into the microplatform simultaneously allowing comparison of their angiogenic potential. Differences in the height and width of created molds and PDMS casts are shown in Figure 4 and Table 1.

#### 3.2.2. Angiogenic Activation

**Figure 5:**
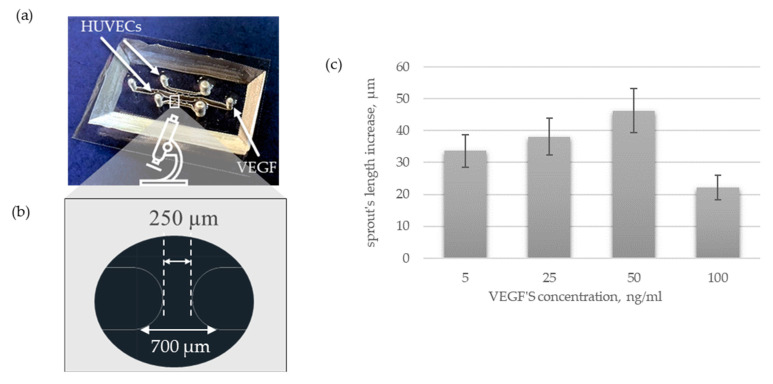

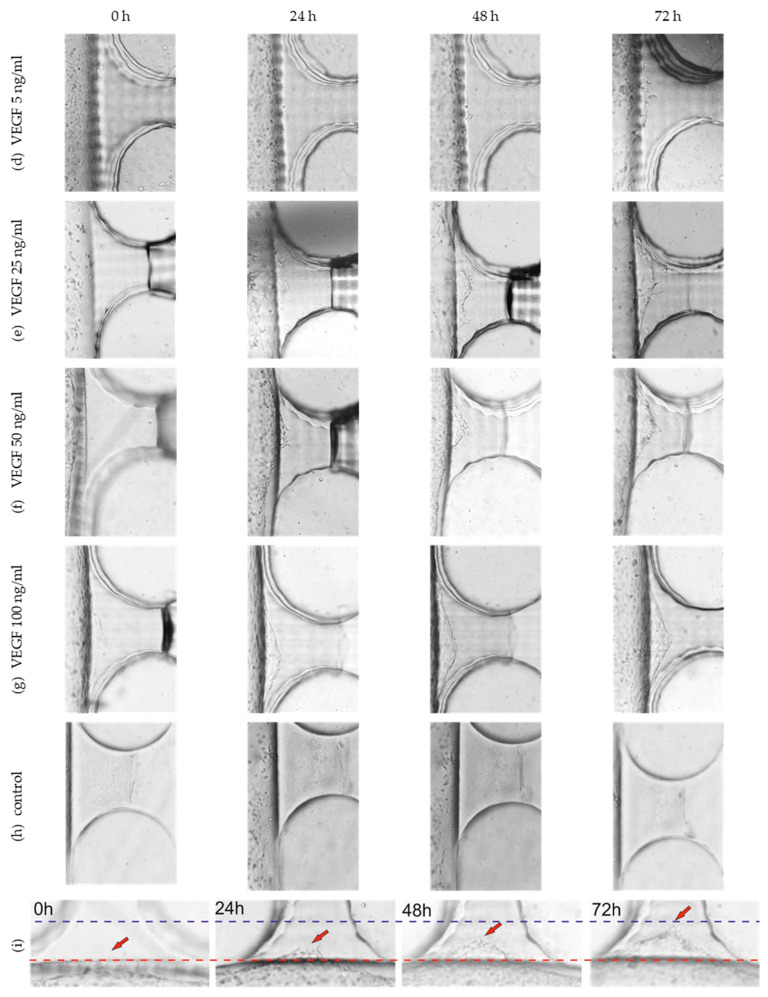
(**a**) Ready-to-use microplatform 3 consisting of PDMS bonded with a coverslip glass. (**b**) Structure of migrating port. (**c**) Quantitative and (**d**–**h**) qualitative analysis over influence of VEGF’s concentration on migration of HUVECs. (**i**) One-factor analysis (VEGF with concentration of 50 ng/mL) of sprout length at different time points. Arrows point to end of sprout. All images were taken with the use of fluorescence microscopy. Scale bar: 100 µm.

Further studies on angiogenic activation with the use of VEGF were performed with the use of microplatform 3 (Figure 4d and Figure 5a). Based on the results obtained for the microplatform with one microchannel, we decided to use droplets with a volume of 2 µL to create lumen within a rectangular microchannel. Those parameters created microvessels smaller than human arteries (360 ± 16 µm), so they can be a good model to study EC spreading. The pro-angiogenic factor was loaded into the central microchannel 24 h after HUVECs seeding. The course of angiogenesis was controlled every day with the use of fluorescence microscopy. As VEGF is well-known for its pro-angiogenic potential it has been introduced to confirm the functionality of our microfluidic culture system. As expected, the migration of ECs was successfully activated. Specifically, first tip cells were observed already after overnight incubation of HUVECs with VEGF. During the next few days, HUVECs progressively migrate within the space of migrating ports forming a dome-shaped sprout. Furthermore, the assessment of the effect of different concentrations of VEGF is poorly standardized and summarized in the literature. Thus, it has been evaluated under consistent conditions as a part of this research. More precisely, the influence of VEGF with concentrations of 5, 25, 50, and 100 ng/mL was examined (Figure 5a). In all cases, explicit migration of endothelial cells has been observed after overnight incubation with VEGF. The number of migrating cells and the length of forming sprouts progressively increase over the following days of cell culture within a microsystem (Figure 5d–g,i). As VEGF is responsible only for the initiation stage of angiogenesis, the most visible change in sprout length has been observed within the first 48 h after its loading. Further migration of HUVECs and lumen formation can potentially be stimulated by the introduction of angiogenic agents such as pericytes. Moreover, quantitative analysis of sprout length increase has been effected. It has been noticed that lower concentrations of VEGF varying from 5 to 50 ng/mL reveal higher angiogenic potential than its higher concentrations, 100 ng/mL in this case. Specifically, in the case of lower values, the following pattern has been observed: the value of sprout length increases slightly with the increase of VEGFs concentration. However, those values do not differ significantly.

Simultaneously with every experiment, HUVECs without any addition of an angiogenic factor have been cultured as a control. In this case, the central microchannel was filled with EGM-2. Migration of HUVECs in those cases (Figure 5g) has not been observed.

## 4. Discussion

Angiogenesis is a complex process that contributes to the development of serious illnesses such as cancers and cardiovascular diseases. A better understanding of mechanisms regulating physiological and pathological angiogenesis is required to design more effective treatments. Recently, fruitful studies on angiogenesis have been realized in microfluidics [32]. Developed microsystems recapitulate chosen combinations of angiogenic factors within selected microenvironments. Subsequent research including missing parameters is needed to engineer a fully functional angiogenesis-on-a-chip microplatform.

First, under physiological conditions angiogenesis is preceded by a vascularization step during which primary vessels with luminal structures are formed. Therefore, it seems essential to provide a cylindrical structure of primary microvessels under microfluidic conditions as well. Predominately, this task is realized with the use of microneedles [23,33,34,35,36,37]. Here, we applied an alternative method which is the modified Viscous Finger Patterning (VFP) technique developed by Bischel et al [31]. It allows an easy application of permanent flow with the use of hoses and a pump. Consequently, VFP can provide an easier analysis of the influence of hemodynamic parameters on the course of angiogenesis in vitro. In contrast to microneedles, VFP enables a reconstruction not only of linear but also of curved and branched vascular geometries [31,38]. Furthermore, VFP is a highly repetitive, fast, simple, and inexpensive technique. Additionally, it allows easy adjustment of lumen diameter. More precisely, Bischel et al. have verified the influence of the pre-incubation time of the collagen matrix on the value of lumen diameter. Information on the effect of the volume of dispensed droplets on lumen diameter was incomplete [31]. Therefore, as part of our research, missing optimization studies were performed. It has been observed that with a decrease in the volume of the droplets, the diameter of the microvessel decreased as well. The faster flow of PBS did result in the production of narrower cylindrical microchannels. Lumen diameter varied from 791 ± 26 µm to 204 ± 23 µm. It has been observed that lumen diameter covers an analogous percentage of rectangular microchannel width for every dosing model. Therefore, mean percentage values for every dosing pattern were determined. Established values allow easy adjustment of the VFP procedure for desired lumen diameter. Thus, lumen diameter can be easily scaled-down following the observed pattern (Figure 2). 

One of the most common agents that determines the initiation of angiogenesis is Vascular Endothelial Growth Factor (VEGF) [39,40]. It has already been successfully used to initiate angiogenesis in microfluidics [20,41,42]. Its concentration can be regulated directly or indirectly. In the case of direct regulation, protein is dosed directly into the flowing medium. It has been observed that its low concentration stimulates ECs migration more efficiently than higher ones. However, it remains unclear what is hidden under the terms “low” and “high”. Some suggest the use of VEGF solution with a concentration of 2.5–5 ng/mL [26] while others with a concentration of 50 ng/mL [22] or even 100–500 ng/mL [23]. As available data are divergent, we have examined the influence of VEGF with concentrations of 5, 25, 50, and 100 ng/mL on the course of angiogenesis under coherent conditions. It turns out that VEGF solution with a concentration varying from 5 to 50 ng/mL reveals similar angiogenic potential while its higher concentration of 100 ng/mL showed poorer pro-angiogenic properties. The greatest value of sprout length increase was observed for a VEGFs concentration of 50 ng/mL (Figure 4b). Furthermore, VEGF can be delivered indirectly by some types of cells such as macrophages, fibroblasts, keratinocytes, platelets, and tumor cells. One of the most often examined co-cultures consists of tumor spheroids and endothelial cells [43,44,45,46,47,48,49]. The pro-angiogenic activity of tumor spheroids can be enhanced by the addition of stromal cells such as fibroblasts [33,37]. Their influence on HUVECs can be analyzed with the use of the designed microplatform.

## 5. Conclusions

In this study, we have designed and manufactured a PDMS/glass microfluidic platform for angiogenesis studies. It includes two cylindrical and perfusable primary microvessels. They were created with the use of Viscous Finger Patterning technique and were subsequently seeded with HUVECs. Their diameter recapitulates the dimensions of vessels smaller than human arteries. Such dimensions provide simple fabrication and lucid microscopic observations. Moreover, we have observed that the diameter of the microvessel decreased with the decrease in the volume of the droplets and that lumen diameter covers an analogous percentage of rectangular microchannel width for every dosing model. Therefore, mean values for every dosing pattern were determined. Established percentage values allow easy adjustment of the VFP procedure for the desired lumen diameter. Thus, lumen diameter can be easily scaled-down following the observed pattern. Homogenous adhesion of HUVECs within the luminal structure was achieved thanks to the double loading of cells accompanied by a single rotation of a microplatform by 180° after the second loading. Furthermore, due to our angiogenesis-on-a-chip microplatform, we have assessed the influence of VEGF solution with different concentrations on HUVECs migration. The spatial arrangement of cells and their morphology were visualized by fluorescence and confocal microscopy. In all cases, HUVECs morphology and behavior were similar. More precisely, we have observed first tip cells after overnight incubation of HUVECs with a selected angiogenic agent. During the next few days, HUVECs progressively migrate within the space of migrating ports forming a dome-shaped sprout. We have further assessed that VEGFs solution with concentration varying from 5 to 50 ng/mL reveals similar angiogenic potential while its higher concentration of 100 ng/mL showed poorer pro-angiogenic properties. All collected data may contribute to the development of more complex vascularized multi-organ-on-a-chip microsystems that could potentially be used for drug screening.

## Figures and Tables

**Table 1:** Comparison of designed dimensions with dimensions of patterned molds. Measurements were executed with the use of 3D Laser Measuring Microscope (LEXT Olympus4000).

			Central	Lateral	Migrating Ports
microplatform 1	lenghth	Designed	1.8 cm	1.6 cm	300.00 µm
		Patterned	not measured.	not measured.	287.07 µm
	height	Designed	1000.00 µm	1000.00 µm	1000.00 µm
		Patterned	998.33 µm	998.33 µm	998.33 µm
	width	Designed	1000.00 µm	1000.00 µm	50.00 µm
		Patterned	1007.22 µm	1025.13 µm	49.12 µm
microplatform 2	lenghth	Designed	0.75 cm	1 cm	500.00 µm
		Patterned	not measured.	not measured.	494.05 µm
	height	Designed	500.00 µm	500.00 µm	500.00 µm
		patterned	496.27 µm	496.27 µm	497.36 µm
	width	Designed	1000.00 µm	500.00 µm	500.00 µm
		patterned	1014.67 µm	503.23 µm	487.64 µm
microplatform 3	lenghth	Designed	1.8 cm	1 cm	500.00 µm
		Patterned	not meausred.	not meausred.	496.72 µm
	height	Designed	1000.00 µm	500.00 µm	250.00 µm
		Patterned	1033.47 µm	505.93 µm	243.30µm
	width	Designed	1000.00 µm	500.00 µm	250.00 µm
		Patterned	979.05 µm	507.26 µm	273.20 µm

## Data Availability

Not applicable.

## Supplementary Materials

The following supporting information can be downloaded at: https://www.mdpi.com/article/10.3390/s22239414/s1, Figure S1: Influence of droplet’s volume on lumen’s diameter., Figure S2: Image of a spinning-wheel used for turn-over of a microplatform.
