# Supplementary material for: Exploring Endothelial Expansion on a Chip
Source: Sensors (Basel). 2022 Dec 2;22(23):9414. doi: 10.3390/s22239414 (PMC9741423; doi:10.3390/s22239414)
Supplement: Supplementary file 1 [file sensors-22-09414-s001.zip › sensors-2024545-supplementary.pdf]

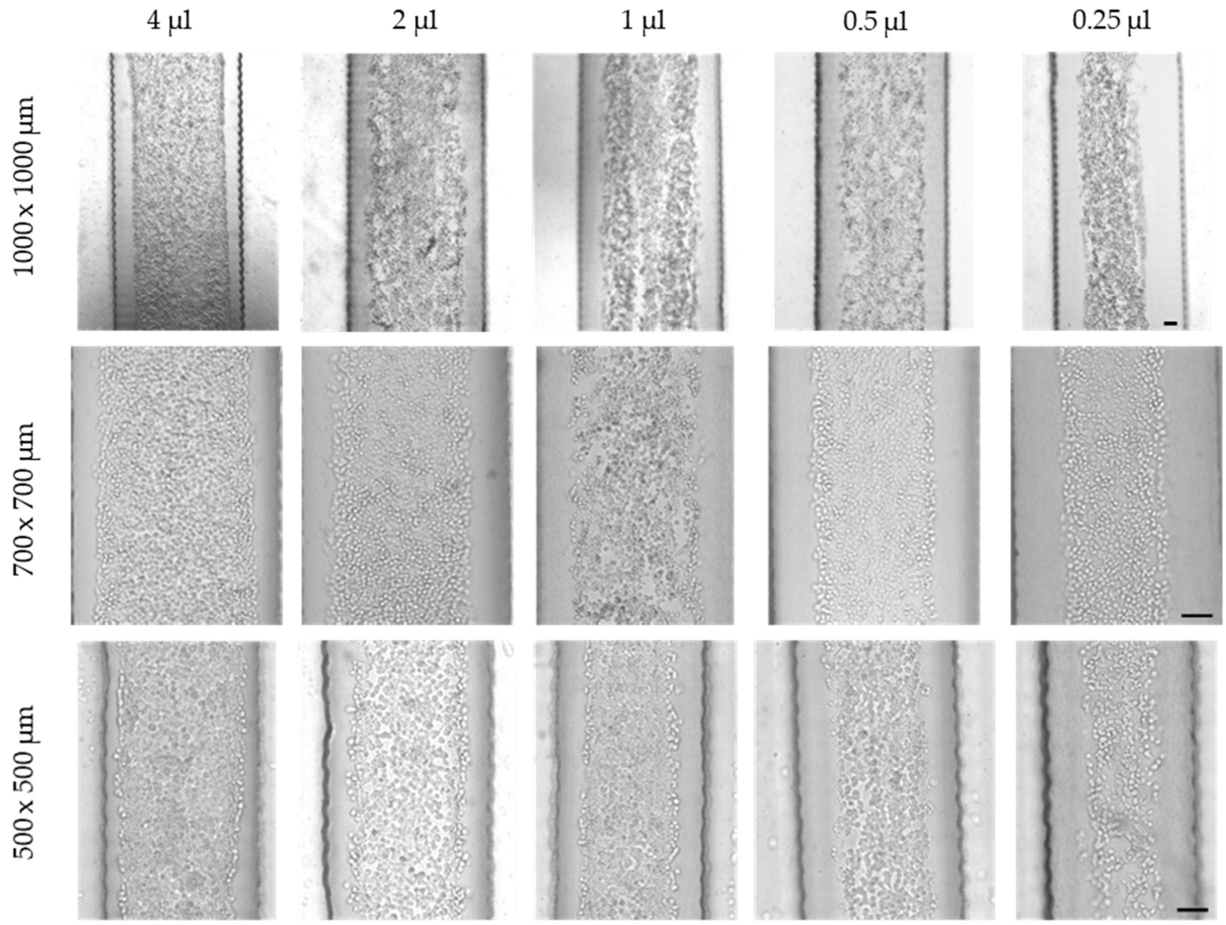

Figure S1. Influence of droplet's volume on lumen's diameter. Scale bar: 100µm.

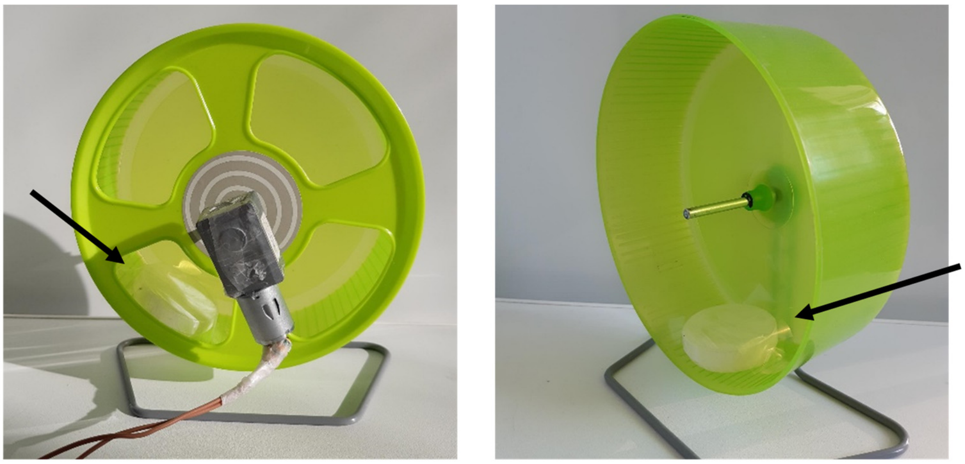

Figure S2. Spinning-wheel used for turn-over of a microplatform. Microplatform is located inside of a Petri dish (black arrows) filled with EGM-2. Another Petri dish containing a microplatform is attached on the opposite side to balance a device.
